# Oxidation of Sperm Nucleus in Mammals: A Physiological Necessity to Some Extent with Adverse Impacts on Oocyte and Offspring

**DOI:** 10.3390/antiox9020095

**Published:** 2020-01-23

**Authors:** Joël R. Drevet, Robert John Aitken

**Affiliations:** 1 Faculty of Medicine, GReD Institute, INSERM U1103—CNRS UMR6293—Université Clermont Auvergne, CRBC building, 28 place Henri Dunant, 63001 Clermont-Ferrand, France; 2 School of Environmental and Life Sciences, Priority Research Centre for Reproductive Sciences, The University of Newcastle, Callaghan, Newcastle 2308, Australia; john.aitken@newcastle.edu.au; 3 Faculty of Health and Medicine, The University of Newcastle, Callaghan, Newcastle 2308, Australia; 4 Medical Genetics, Hunter Medical Research Institute, New Lambton Heights, 13 2305 Newcastle, Australia

**Keywords:** spermatozoa, nuclear integrity, oxidative DNA damage, putative transgenerational impacts

## Abstract

Sperm cells have long been known to be good producers of reactive oxygen species, while they are also known to be particularly sensitive to oxidative damage affecting their structures and functions. As with all organic cellular components, sperm nuclear components and, in particular, nucleic acids undergo oxidative alterations that have recently been shown to be commonly encountered in clinical practice. This review will attempt to provide an overview of this situation. After a brief coverage of the biological reasons why the sperm nucleus and associated DNA are sensitive to oxidative damage, a summary of the most recent results concerning the oxidation of sperm DNA in animal and human models will be presented. The study will then attempt to cover the possible consequences of sperm nuclear oxidation on male fertility and beyond.

## 1. Oxidative Stress

Oxidative stress is inherent in the consumption of oxygen by aerobic organisms that metazoans have made their “fuel” for the production of energy via the cellular mitochondrial respiratory chain. In doing so, cells produce active oxygen derivatives commonly referred to as reactive oxygen species (ROS) which include free radicals (such as the superoxide anion O_2_∙^−^ and the hydroxyl radical OH∙) and non-radical molecules such as hydrogen peroxide (H_2_O_2_). These molecules are unstable and propagate instability by trying to capture a stabilizing electron, which leads to the oxidation of other molecules which, in turn, seek other targets [1].

Even if lipids are the most sensitive organic components to oxidation, none of them escape it, proteins, sugars, and nucleic acids also being involved. To fight against oxidative attack, cells of aerobic organisms have developed a set of countermeasures in the form of small molecules that are capable of trapping free radicals (glutathione, thioredoxin, vitamins, polyamines, polyphenols) and antioxidant enzymes (superoxide dismutase (SOD), glutathione peroxidases (GPxs), Catalase, peroxiredoxins (PRxs), glutaredoxins (GRx)) [2,3] that intervene intra- and extracellularly to regulate the presence of reactive oxygen species (ROS).

**Figure 1:**
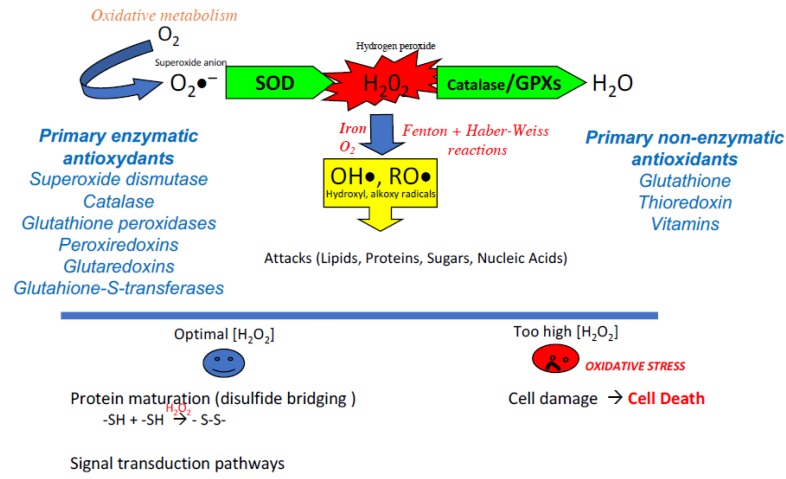
The classic reaction cascade for the production of reactive oxygen species of metazoans. The consumption of oxygen, which supports the production of energy by the mitochondrial respiratory chain, results in the production of the anion radical superoxide (O_2_∙^−^). Although weakly reactive and not permeant the superoxide anion is readily transformed (via the action of superoxide dismutase: SOD) into an active oxygen derivative, hydrogen peroxide (H_2_O_2_). This molecule occupies a crossroad position having important actions in the maturation of proteins via the oxidation of thiol groups as well as serving as a stimulus and second messenger in critical signal transduction pathways. However, if an excess of H_2_O_2_ is produced, its strong penetration into the cellular compartments and its rapid reactivity with iron and oxygen (via the Fenton and Haber-Weiss reactions) lead to the production of very aggressive free radicals (hydroxyl and alkoxy radicals) for which there is no active recycling system. These free radicals attack all organic components (lipids, proteins, sugars, and up to nucleic acids). The multiple alterations generated if they are not sufficiently corrected can lead to cellular dysfunctions and eventually to cell death. To finely regulate the concentration of H_2_O_2_, both in the extracellular and intracellular compartments, metazoans have developed several enzymatic activities grouped under the classification of primary enzymatic antioxidants (GPxs: glutathione peroxidases, CAT: catalase, PRxs: peroxiredoxins, GRx: glutaredoxins, GSTs: glutathione S-transferases) to transform H_2_O_2_ into a neutral element, water (H_2_O). In the same way, several non-enzymatic molecules (glutathione, thioredoxin, vitamins) are at work to trap free radicals.

Although ROS have long been considered as aggressors leading to cell death and pathophysiology, their important physiological actions should not be neglected [4]. Indeed, ROS are regulators of cellular activity, acting essentially as second messengers and participating in the physiological oxidation of cellular components [5,6]. H_2_O_2_ is an ROS at the crossroads, both an intra- and extracellular signaling molecule, a powerful bactericidal agent also acting as an important player in our inflammatory/immune responses [7,8], and an essential factor in the disulfide bridging of proteins carrying thiol groups [9], whether spontaneously or mediated upon by enzymes (see Figure 1).

The balance between the production and recycling of ROS is, therefore, a key element of cell homeostasis. An excess of ROS (whether due to overproduction or lack of recycling) can then lead to a situation known as “oxidative stress.” This is accompanied by a set of alterations to cellular components, which affect cellular structures and functions and ultimately lead to cell death (Figure 1). Although oxidative stress is now recognized as a component of cell dysfunction, pathophysiology, and aging, it is important to note that its opposite, reductive stress (not enough ROS and/or too much antioxidants), is just as problematic for cell homeostasis. The notion of redox balance underlines this dual aspect. What are the situations in which this redox balance is called into question?

**Figure 2:**
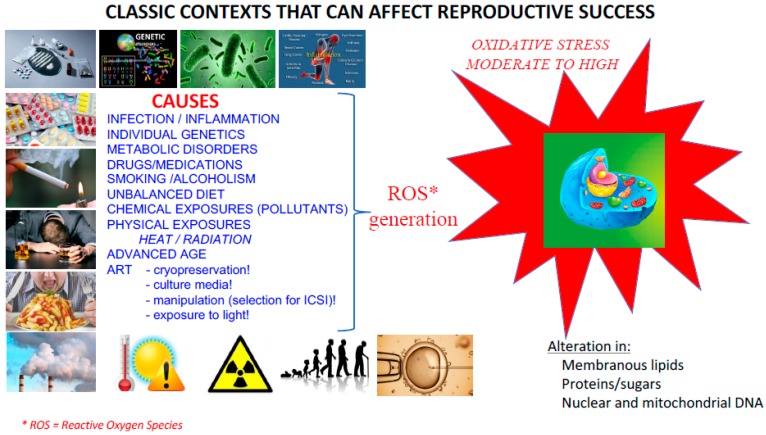
Classic situations promoting the generation of ROS. Situations as varied and cumulative as individual genetic predisposition, infectious/inflammatory pathophysiology or metabolic disorders, medication or addiction (drugs, alcohol, tobacco), nutritional imbalances, exposure to environmental pollutants and physical stresses (such as excessive heat, ionizing radiation) all generate systemic or local ROS that can affect all cells of the body including gametes. Aging is also a situation that promotes oxidative stress. Indeed, according to the “free radical theory of ageing”, the lower efficiency of ROS recycling systems when one ages leads to an inevitable increase in ROS production. Finally, in the very specific context of assisted reproductive technologies (ART), gamete cryopreservation, culture in different artificial media, long gamete selection protocols for in vitro fertilization, intracytoplasmic sperm injection (IVF ICSI) and exposure to light alone are all sources that can lead to a pro-oxidant situation.

The answer is both easy and complicated in that almost all of a mammal’s interactions with its environment are likely to generate both local and systemic oxidative stress (see Figure 2 for an illustration of the classic causes that can lead to overproduction of ROS). Most cells interpret this ROS signaling, whether internal and/or external, use it and/or fight it in a very refined and effective manner. However, the mature male gamete, for multiple reasons discussed below, has some gaps in its management of ROS that make it particularly vulnerable [10]. 

## 2. The Spermatozoon’s Particular Susceptibility to Oxidative Insults 

**Figure 3:**
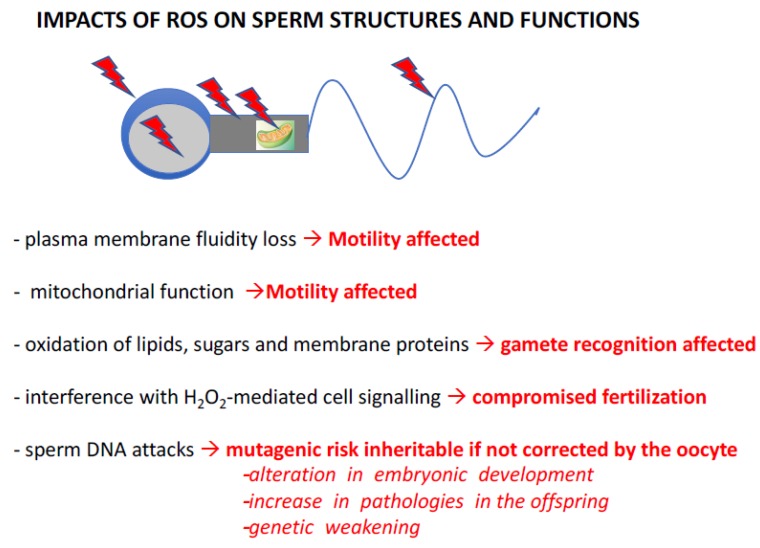
Known impacts of ROS on sperm structures and functions. ROS (red arrowheads above and within the schematized spermatozoa) have detrimental effects on sperm cells, resulting in changes in their structure and function. Peroxidation of membrane lipids affects the fluidity of the lipid bilayer, resulting in changes in sperm motility. Similarly, oxidative damage to enzyme complexes in the mitochondrial respiratory chain leads to changes in sperm motility. In an entirely different register, damage to the plasma membrane, whether from lipids, sugars and/or transmembrane proteins incorporated into the lipid bilayer, can alter the spermatozoa’s ability to interact with their target, the oocyte. At the same time, ROS (especially H_2_O_2_) can disrupt the terminal signaling pathways of capacitation and acrosomal reaction, thereby disrupting the fertilization stage. Finally, by attacking nucleic acids, ROS induce a mutagenic risk that can be transmitted to the embryo and future generations if these alterations in the genetic and epigenetic information contained within the paternal nucleus/DNA are not effectively corrected by oocyte repair systems. These de novo mutations, created in the female germline but originating in the male, then increase the risk of abnormal development, the appearance of pathologies in the offspring and, in the long term, may lead to the genetic impoverishment of the species. Please refer to articles [13,14,15] for appropriate literature covering these aspects.

Several characteristics of spermatozoa explain their sensitivity to oxidative stress. If most cells fight oxidative stress by the presence of small molecules and the activity of antioxidant enzymes contained in their cytoplasm and/or, if necessary, by transcriptional activation of the genes corresponding to these proteins, then spermatozoa are the exception. Evolution has chosen, in internally fertilized metazoans, to produce an extremely cyto-differentiated male gamete characterized by the exclusion of most cytoplasm and the significant compaction of its genetic material, making this silent cell unable to transcribe and synthesize new proteins. The “silent” nature of this cell also explains its inability to repair the alterations that affect it, and in particular, it cannot repair the damage to its genetic material. Death by necrosis or apoptosis is the only alternative for this cell if submitted to acute stressors. As a result, spermatozoa are unable to defend themselves effectively against oxidative stress. As a consequence, in their post-testicular life, these cells largely depend on their immediate environment and their very particular organization (highly compacted nucleus) for protection. This situation of fragility in relation to oxidative stress is also aggravated by the peculiar lipid composition of the plasma membrane of the spermatozoon. Of all the differentiated cells in a mammal, spermatozoa contain the highest level of polyunsaturated fatty acids (PUFAs) in their membranes [11], which are the main targets of ROS. The oxidation of these PUFAs generates toxic aldehydes which, in a vicious cycle, amplify the production of ROS [12] and their pathological consequences. Figure 3 illustrates this in a very schematic way (as it is not the focus of the present review) capturing the known and suspected consequences of oxidative stress on sperm structures and functions. In summary, peroxidation of membrane lipids will affect sperm motility by altering the fluidity, and therefore flexibility, of the membrane, which are important factors for flagellar movements. Sperm motility will also be hampered by the loss of efficiency of mitochondria when subjected to oxidative stress. In addition, the oxidation of membrane lipids and transmembrane proteins incorporated in the lipid bilayer will affect both spermatozoa-oocyte interaction and the signaling cascades resulting from this event. This will lead to poor spermatozoa/oocyte recognition as well as altered capacitation and acrosomal reaction processes, which are crucial steps for successful fertilization [13,14,15].

**Figure 4:**
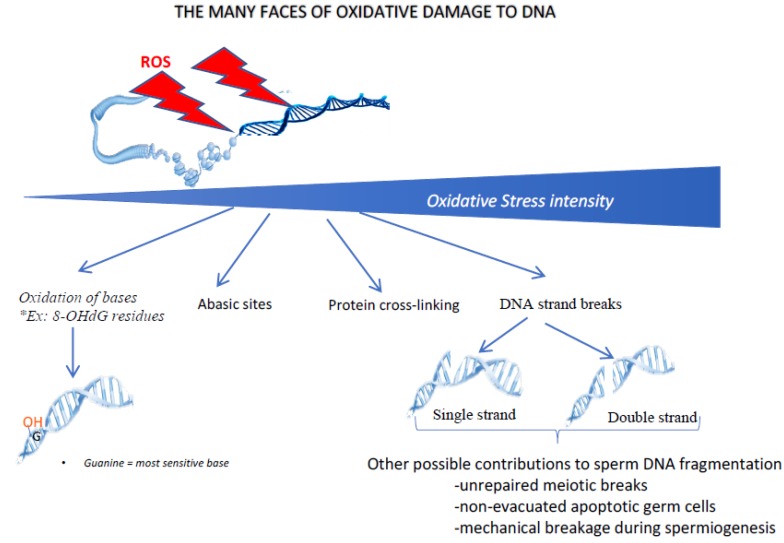
The oxidative damage of DNA has many faces. Depending on the intensity of oxidative stress, different types of nuclear/DNA alterations can be observed. In the case of low/light oxidative stress, the first type of damage concerns the oxidation of DNA bases (in particular guanosine and adenosine) leading to oxidized residues (such as 8-oxo-guanosine, the so-called 8-OHdG residue). When the level of oxidative stress increases, other alterations can occur, including the generation of abasic sites, cross-link of nuclear proteins and rupture of DNA strands, either single or double. Although DNA strand breaks may have an oxidative origin, it should be kept in mind that the fragmentation of sperm DNA may have other origins, including unrepaired meiotic breaks, non-processed apoptotic germ cells or mechanical shearing during late spermatogenesis (spermiogenesis) when protamines replace sperm nuclear histones.

Last, but not least, one of the major consequences of oxidative stress on sperm cells involves damage to the paternal genetic material. Figure 4 shows some of the multiple ways in which oxidative stress can damage the cell nucleus and its contents. Depending on the intensity of the oxidative stress, it can range from simple oxidation of bases (guanosine and adenosine being the most sensitive bases to oxidative stress) to DNA fragmentation (by single- or double-strand breaks). In between, other DNA oxidative events can be found, including the generation of abasic sites and DNA-protein cross-linking. As mentioned above, as mature sperm cells lack a fully functional DNA repair system, they will need to rely on oocyte DNA repair systems (mainly the post-fertilization oocyte base excision repair pathway, BER) to correct these oxidative alterations. Even in situations of moderate to low oxidative stress, which will not cause DNA breakdown, oxidation of the bases will occur and must be corrected (i.e., each oxidized base must be replaced by a non-oxidized base).

## 3. Oxidation Processes are Required for Optimal Maturation of Sperm

Although, as mentioned above, sperm structures and functions are easily threatened by oxidative alterations, oxidative processes contribute to the production of fully mature sperm cells. In the late 1980s, it was elegantly reported that during post-testicular maturation of sperm cells (i.e., during epididymal maturation), sperm proteins undergo high disulfide bridging activity [16]. In short, if we look at the level of thiol groups carried by sperm proteins in the caput epididymis, we see that it consists mainly of free thiols, whereas in spermatozoa from the caudal epididymis, most free thiols have been converted into disulfide bridges. Disulfide isomerases are at work in the epididymis to create these bridges by using hydrogen peroxide as an oxidizing agent. Many sperm proteins feature disulfide bridges, whether they are part of the plasma membrane or more internal; regardless of their location in the head, midpiece, or tail segment of the spermatozoon. It is assumed that these intra- and/or inter-protein interactions are created for mechanical purposes, stiffening key structures within the spermatozoon and making the latter more resistant to attack. It is also suspected that these finely tuned protein-protein interactions involving disulfide bridges could determine optimal flagellar movement and motility of sperm cells. In the nucleus, this disulfide bridging activity is also at play and concerns cysteine-rich protamines. We and others have helped to show that an enzymatic activity contained in the sperm nucleus (snGPx4, for sperm nucleus glutathione peroxidase 4) was actually a disulfide isomerase using luminal hydrogen peroxide in the caput epididymis to make inter- and intra-protamine disulfide bridges that further condense the sperm nucleus and lock it in an optimally compacted state during epididymal transit [17,18,19]. This was further reinforced by the observation that when the luminal hydrogen peroxide concentration of the epididymis was modified [19,20], it immediately resulted in a higher transient condensation of the sperm nucleus in the caput epididymis [20,21], followed rapidly by excessive oxidative alterations of the DNA due to both the ability of H_2_O_2_ to cross the plasma membrane and the difficulty spermatozoa experience in defending themselves against such attacks. Finely controlled post-testicular oxidation processes are therefore at work to define an optimal state of nuclear condensation of the sperm nucleus. This is of paramount importance as the high compaction of paternal DNA is one of the means chosen by evolution to protect sperm DNA from mutagenic alterations. The process is finely balanced because not all free thiols of cysteine-rich protamines are affected by disulfide bridging. Some thiols are associated with zinc, thus preventing a number of them from being involved in disulfide bridges. It is therefore assumed that it is important not to over-condense the already highly compacted sperm nucleus so that the oocyte does not have too much difficulty post-fertilization, in creating the male pronucleus, a process that has recently been shown to be controlled by thiol-reducing activity [22]. This beneficial post-testicular oxidation process illustrates very well the Jeckyl and Hyde act played by ROS on sperm structures and functions and in particular on the nucleus.

The fact that it has long been reported that sperm cells are themselves very good producers of ROS [23], a logical consequence of their high mitochondrial activity when they are motile, is ambiguous. Could evolution have had the opportunity to do otherwise? Certainly not! Sexual reproduction requires the physical union of male and female gametes, so one or both cells must be capable of movement to achieve fertilization. Movement requires energy, which is supplied to aerobic organisms by oxygen consumption via the mitochondrial respiratory chain, inevitably leading to the generation of ROS. Thus, ROS are an inevitable consequence of sperm movement. However, evolution has been smart enough to dampen the harmful effects of ROS by grouping mitochondria into a well-defined subcellular compartment, the sperm midpiece, where some of the free radicals can be neutralized before they have a chance to enter the nucleus.

## 4. Sperm DNA Oxidation is Conditioned by the Chromatin Organization

**Figure 5:**
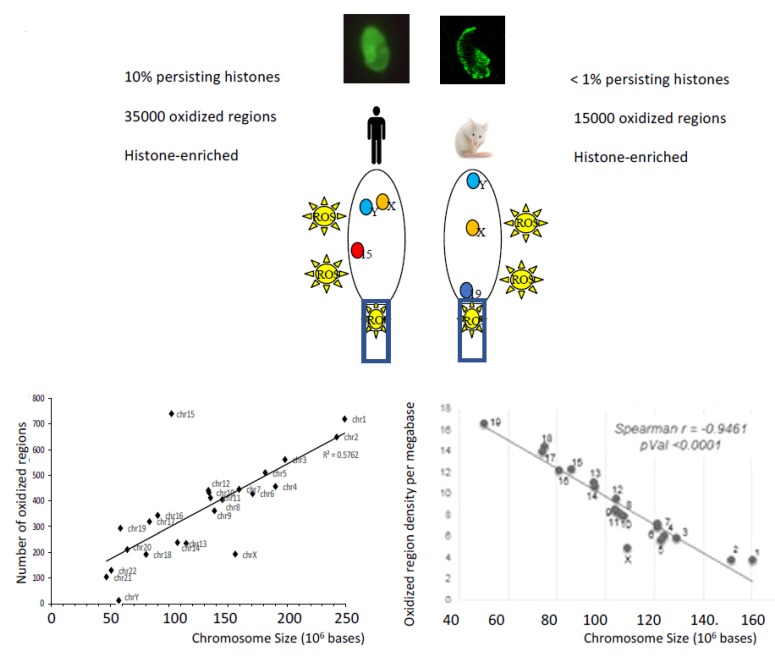
The nuclear regions sensitive to oxidation depend on the chromatin organization of the sperm cells, which is species-specific. In humans and mice, DNA oxidative alterations of spermatozoa revealed by the presence of 8-OHdG residues by fluorescent confocal microscopy show distinct patterns. The low nuclear condensation state of human spermatozoa in which a large part of the chromatin is still in a nucleosomal organization (i.e., associated with persistent histones) explains why 8-OHdG residues are found throughout the sperm nucleus and why there are so many oxidized domains. This low state of nuclear compaction is also illustrated by the observation that the number of oxidized regions follows a linear relationship with the length of the chromosomes. In the more compacted mouse sperm nucleus containing few histone-bound nucleosomes, the regions sensitive to oxidation are more peripheral and less numerous. In this less accessible context, the linear relationship between chromosome length and the number of oxidized regions is no longer valid and only the most exposed (peripheral) chromosomes are affected by oxidative alterations (as illustrated by autosome 19 and chromosome Y [21,25]). In both species, the more chromosomes are located at the periphery of the sperm nucleus the more sensitive they will be to oxidative DNA damage [21,25,26]. In both species, oxidized nuclear regions are regions enriched with persistent histones [21,25,26].

We have already mentioned above, that whenever ROS homeostasis is modified around sperm cells, there is a risk of excessive oxidation that can affect its structures and functions. In this section, we will focus on the oxidation of sperm DNA and, in particular, where it occurs in the mammalian sperm nucleus from recent data coming from animal and human studies [21,24,25,26]. We have shown that the peripheral regions of the mouse sperm nucleus are more sensitive to oxidative DNA alterations [24]. We also showed that the basal region of the sperm nucleus in the immediate vicinity of the sperm midpiece, the internal source of ROS, was another area preferably affected by oxidative damage [24]. Given the notion of chromosomal territories (CT) within spermatozoa (referring to the fact that within the sperm nucleus, chromosomes are not randomly organized but occupy specific positions identical from one sperm cell to another [27]), it was logical to find that the chromosomes that were most sensitive to oxidative damage were those located in the nuclear periphery and at the base of the sperm nucleus [25]. In the case of the mouse sperm nucleus, this referred to the small autosomes (Chr19, Chr18 and Chr17), all three of which are located near the neck of the cell, closer to the midpiece (see Figure 5). This also concerned the Y chromosome, which occupies a particular position in the murine sperm nucleus near the thin, hook-shaped apical head region [21,25]. In addition, it was very logical to find that regions of low compaction within chromosomes (corresponding to regions maintained in a nucleosomal organization, i.e., still associated with persisting histones that were not replaced by protamines during spermiogenesis) were particularly sensitive to oxidative attack [25]. This conclusion was strongly supported by confocal microscopic images showing that the oxidized regions of the murine sperm nucleus fully corresponded to the nuclear domains enriched with persistent histones [24]. In addition, we further observed that the small DNA regions (about 1 kb long) connecting one protamine toroid to the next one (the interlinker regions) were systematically more sensitive to oxidation along each chromosome than the domains associated with protamines [25]. This has been illustrated by the rhythmic presence of oxidized DNA domains on the chromosomes at about 50 kb intervals, which corresponds exactly to the length of DNA associated with each protamine toroidal ring [25]. Therefore, paternal DNA regions of easy access, lower condensation and near ROS sources (external/internal) are those that will preferably undergo oxidative alterations.

Identical investigations conducted on human spermatozoa confirmed these observations [26]. In the nucleus of human spermatozoa, the chromosome regions sensitive to oxidation concern the peripheral nuclear territories and the chromosome domains associated with histones [26]. The same rhythmic pattern of oxidized regions occurring approximately every 50 kb on the chromosomes and corresponding to the inter-toroid DNA linkers has also been observed in the human sperm nucleus [26]. The only difference between the chromosome regions of the mouse and human sperm nucleus sensitive to oxidation comes from the observation that in the human sperm nucleus, almost all chromosomes were fairly equally affected by oxidative damage [26]. This led to the observation of a linear relationship between the number of oxidized regions and the length of each chromosome. This was not the case in the highly compacted mouse sperm nucleus where small chromosomes because of their localization at the nuclear periphery were most affected by oxidative damage [25]. This difference between the two species is easily explained by the fact that the human sperm nucleus retains a very high proportion of persistent histones, compared to the mouse sperm nucleus, which makes it much less condensed [28]. Naturally, this structural feature is likely to facilitate oxidative damage along the length of each human sperm chromosome. Following the same logic, the number of regions sensitive to oxidation in the weakly condensed chromatin of human spermatozoa was much higher than that of a mouse sperm cell (see Figure 5). However, this comparison is of limited value and may be purely fortuitous as it is difficult to compare one model with the other. Indeed, it is unlikely that the basal oxidation level of spermatozoa from a normozoospermic human donor can be considered equivalent to the basal oxidation level of wild-type (WT) mouse sperm cell. Despite the more homogeneous distribution of oxidized regions on the chromosomes of the human sperm nucleus, some chromosome domains have nevertheless proved to be particularly sensitive to DNA oxidation [26]. Such an example is the q11–q14 domain of chromosome 15 of the human sperm nucleus where 3 regions of susceptibility to oxidation have been found. Interestingly, one of these regions (q13–q14) overlaps a locus in which are located genes involved in syndromes whose frequency in offspring has been associated with the age of the father and sperm DNA lesions. These syndromes were also associated with poor DNA repair activities in the oocyte, probably related to maternal age [26]. Contrary to what might have been expected by looking at the repetitive and G-rich signature of the telomeres (5’-TTAGGG-3’), we did not observe any particular sensitivity to telomere oxidation in any of the models studied (human [26] or mouse spermatozoa [24]). Although this may have been due to the technical limitations associated with sequencing regions full of repetitive sequences, this observation is not conducive to a strong impact of oxidation on the length of spermatozoa telomeres. Despite reports showing that short telomeres are associated with sperm DNA alteration (mainly fragmentation) and male infertility [29,30], it is not yet clear that DNA oxidation directly influences spermatozoa telomere length [31,32]. A recent report suggests, however, that ROS may inhibit telomerase activity [33] thus indirectly explaining why male infertility cases may be found characterized by spermatozoa with shorter telomeres.

## 5. Consequences of Sperm DNA Oxidation

In the transgenic *gpx5*^−/−^ mouse model (characterized by a light oxidative epididymal luminal environment [20]), it is estimated that more than one million oxidized guanosine residues (so-called 8-oxodG residues) distributed in 16,000 regions on the chromosomes have occurred, of which 1000 are particularly oxidized (hot spots [25]). Considering that two of the four deoxynucleotides (guanosine and adenosine) are sensitive to oxidation, this gives an idea of the extent of oxidative DNA damage that a sperm nucleus can undergo. One of the interesting aspects of this mouse model is that the oxidation of sperm DNA is not associated with DNA fragmentation [20], two conditions that are widely confused in both the clinical and scientific communities. Indeed, while it is true that massive oxidation of sperm DNA can lead to DNA fragmentation, fragmentation of sperm DNA can also result from unrepaired meiotic arrests, poor elimination of apoptotic germ cells and mechanical shearing of the sperm nucleus during the protamination process of spermiogenesis. Thus, DNA fragmentation should not automatically result in the oxidation of sperm DNA. Conversely, the absence of DNA fragmentation does not necessarily mean the absence of DNA oxidation as moderate oxidation does not create single and/or double-strand breaks. This finding, which has not yet percolated clinically, is very well illustrated by the *gpx5*^−/−^ mouse model, whose phenotype is only a slight oxidation of the sperm DNA without an increase in DNA fragmentation [20]. Nevertheless, when knockout (KO) males were mated with WT females of proven fertility, an increase in abortions, abnormal developments and perinatal mortality were recorded compared to WT/WT crosses [20]. This clearly shows that even a mild oxidation of the sperm DNA alone is sufficient to trigger reproductive failures. As the fertilizing capacity of these spermatozoa with an oxidized nucleus is not affected in any way, reproductive failures could only result from an aberrant repair of paternal DNA by the oocyte. It is the oocyte’s task to ensure that the oxidized residues of the paternal nucleus are removed during the decondensation and de-protamination steps following fertilization. This will bring the paternal nucleus into a pronuclear state facilitating syngamy and the restoration of diploidy.

It has been shown that the sperm nucleus contains only the first step of the base excision repair pathway (BER) represented by the activity of 8-oxoguanine DNA glycosylase 1 (OGG1 [34]). It appears that sperm do not have the other components of the BER pathway, which includes *apurinic endonuclease 1* (APE1) and *X-ray repair complementing defective repair in Chinese hamster cells 1* (XRCC1) activities [34]. Only the oocyte BER system can complete the repair process using its XRCC1/APE1 activities to engineer the replacement of oxidized bases with non-oxidized residues. This oocyte-driven DNA repair process underlines the importance of oocyte quality in preventing transmission of paternal DNA alterations into the embryo. Whenever the oocyte’s ability to repair is reduced (e.g., due to maternal age or non-physiological pressure linked to forced oocyte production following hormonal stimulation during the course of ART IVF procedures [35]), there is a risk that unrepaired oxidized residues remain in the paternal pronucleus. If the oocyte BER pathway is not fully effective, and/or if the level of oxidized bases in the sperm nucleus is too high to be treated properly, this will result in nonrepair and/or false repair leading to mainly transversion-type mutations (following Hoogsteen base pairing between 8-OHdG and adenine [36]) that will ultimately be transmitted to the developing embryo and future generations [37]. Depending on where this occurs, it may call into question the completion and/or normality of embryonic development as well as the quality of life of the future individual and beyond. In addition, unrepaired 8-OHdG in paternal pronucleus could, especially if they occur on CpG islands, have an impact on the reprogramming of the methyl epigenetic mark of adjacent cytosines. Indeed, it has been shown that oxidized guanine suppresses the methylation of an adjacent cytosine [38,39,40]. This could lead to aberrant DNA methylation in the embryo as remethylation of the paternal nucleus occurs after fertilization [41]. Aberrant methylation of embryonic DNA may explain abnormal development, altered gene expression, genomic instability and the susceptibility of offspring to disease [42].

## 6. Sperm Nuclear Oxidation May Go Well Beyond Base Alterations

If sperm DNA is sensitive to oxidative attacks, this is also the case for the other components of the nuclear compartment, i.e., the nuclear proteins and the recently characterized nuclear complement of non-coding RNA (ncRNAs). Together with methylation of cytosine residues, nuclear protein modifications and the ncRNA profile represent the three levels of epigenetic information carried by the spermatozoon.

Spermatozoa cytosine hypomethylation has been associated with infertile patients with oxidative DNA damage (mainly DNA fragmentation) and elevated seminal ROS; a situation that has been corrected by antioxidant supplementation [43]. It has been suggested that glutathione synthesis and homocysteine recycling via the single carbon cycle are the pathways linking oxidative stress and cytosine hypomethylation [44]. The oxidation of DNMTs (DNA methyltransferases) decreasing their activity and, as indicated above, the lower cytosine methylation in oxidized CpG regions, are other pathways by which oxidative stress can influence sperm DNA methylation [45]. Modification of the sperm cytosine methylation profile by oxidation is an important issue that deserves the attention of the clinical and scientific communities, as it may be closely related to environmental exposures and ART [46,47].

In addition to the impact of an oxidized G residue on the methylation process, there is a second question to consider theoretically if a post-testicular oxidative stress situation occurs. It concerns the oxidation of methylcytosine (meC) residues carried by the spermatozoa to create hydroxymethylcytosine residues (hmeC). It is interesting to note that the generation of hmeC is the first step in an enzymatically-mediated oxidation process (via the TET enzymes: Ten of Eleven Translocases) which is used to remove meC marks [48] during the post-fertilization reprogramming of the male pronucleus. This is particularly important for the male pronucleus because the sperm nucleus has been highly methylated during spermatogenesis. However, it has been shown that some regions of the male pronucleus must escape this meC erasure process and are therefore maintained in a silent transcriptional state [49]. If these regions are not properly hydroxymethylated in an oxidation process independent of TET, this could lead to the demethylation of paternal genomic regions that should normally be methylated. Such events could lead to significant changes in the embryonic epigenetic fingerprint later in development. Experiments are underway to test this hypothesis. Preliminary data suggest that a post-testicular pro-oxidant environment alters both meC and hmeC distribution within the sperm nucleus [50] In the genetic contexts of WT and *gpx5*^−/−^ mice, we are presently carrying out sperm chromatin immunoprecipitation experiments using antibodies specific for meC and hmeC to identify chromatin regions subjected to differential methylation/hydroxymethylation of cytosine residues.

Sperm nuclear proteins can also be affected by oxidative alterations as is the case for any protein. Protein oxidation essentially results in protein carbonylation [51] and redox thiol modification. Protein carbonylation is defined as the covalent and irreversible modification of the side chains of the amino acids cysteine, histidine and lysine by peroxidized lipid intermediates such as 4-hydroxy, 4-oxoneonenal (4-HNE) [52]. As noted above, there is considerable evidence that oxidation of sperm nuclear proteins containing thiols (including protamines) affects the structure and function of sperm cells. Besides thiol-oxidation, the carbonylation of sperm protamines can occur in a pro-oxidant environment because the protamines are rich in cysteine, histidine and lysine residues. It is not expected to be particularly damaging to the embryo as the nuclear protamines in the sperm are quickly removed after fertilization and replaced by histones. It is, however, possible that protamine carbonylation, advanced glycation end products (AGE) and other sperm nuclear protein-protein cross-linking events that are facilitated upon protein oxidation might modify the kinetics of this protamine replacement process, which was recently shown to be redox-mediated in the oocyte [22]. More important is probably the oxidative alteration of the persistent nuclear histones in spermatozoa. To date, it is understood that these paternal histones will not be replaced in the oocyte and will, therefore, be transmitted to the developing embryo. If oxidative alterations occur in these histones, this could create unsuspected problems in the developing embryo, as these histones will be part of the zygote histone code. To our knowledge, this particular area has not yet been investigated.

Over the past decade, there has been considerable evidence that sperm cells provide the oocyte with a complex and highly dynamic load of non-coding RNA (ncRNA), which represents another aspect of the paternal epigenetic heritage. Two recent studies have shown that environmental constraints such as a particular diet or exposure to behavioral stress modify the profile of sperm RNA [53,54]. These studies also showed unequivocally that these different sperm ncRNA contents were responsible for the transmission of the paternal phenotype to the offspring. In addition, it has been shown that the apocrine secretory activity of the epididymis (i.e., via epididymosomes) is the source of these changes. We have very recently contributed to this area by showing that the distribution of ncRNAs of the epididymal epithelium is significantly altered when WT mice are compared to the pro-oxidant situation experienced by *gpx5*^−/−^ KO mice [55]. As the epididymis is a provider of ncRNA during sperm transit, it is therefore expected that if the ncRNA profile of the epididymis changes, the sperm ncRNA profile will also change. We are currently comparing the ncRNA content of sperm from WT mice versus *gpx5*^−/−^ Not surprisingly, our preliminary results [56] confirm this hypothesis. How and to what extent these different sperm ncRNAs can affect the embryo development program and the health of the offspring are investigations that must now be conducted. 

## 7. Conclusions

Whether they are unrepaired or falsely repaired oxidized bases promoting the creation of de novo mutations in the embryo, multiple epigenetic alterations (whether due to changes in the methylation/hydroxymethylation status of sperm cytosines, the paternally-associated histone code and/or the sperm ncRNA profile), it is obvious that oxidative stress will have a profound impact on the sperm structures and functions as well as on the messages it carries in the oocyte and embryo. These are important questions that still need to be better assessed, especially with regard to the clinical consequences of ART. As sperm DNA oxidation is much more common in infertile patients than sperm DNA fragmentation, a better understanding of its consequences on the embryo and the future individual is needed. In particular, artificial oxidative situations generated in the context of ART should be seriously evaluated, as they may partly explain the low success rate associated with this technology, which has not improved significantly over the past 25 years.
